# Implications From the Analogous Relationship Between Evolutionary and Learning Processes

**DOI:** 10.1002/bies.70027

**Published:** 2025-06-08

**Authors:** Jason Cheok Kuan Leong, Masaaki Imaizumi, Hideki Innan, Naoki Irie

**Affiliations:** ^1^ Research Center for Integrative Evolutionary Science (RCIES) SOKENDAI Hayama Kanagawa Japan; ^2^ Komaba Institute of Science, The University of Tokyo Meguro Tokyo Japan; ^3^ RIKEN Center for Advanced Intelligence Project Chuo Tokyo Japan

## Abstract

Organismal evolution is a process of discovering better‐fitting phenotypes through trial and error across generations. This iterative process resembles learning processes, an analogy recognized since the 1950s. Recognizing this parallel suggests that evolutionary biology and machine learning can mutually benefit from each other; however, ample opportunities for research into their corresponding concepts remain. In this review, we aim to enhance predictive capabilities and theoretical developments in both fields by exploring their conceptual parallels through specific examples that have emerged from recent advances. We focus on the importance of moving beyond predictions by machine learning approaches for specific cases, but instead advocate for interpretable machine learning approaches for discovering common laws for predicting evolutionary outcomes. This approach seeks to establish a theoretical framework that can transform evolutionary science into a field enriched with predictive theory while also inspiring new modeling and algorithmic strategies in machine learning.

## Graphical abstract

Organismal evolution resembles learning processes in many aspects, but the analogy has not been widely recognized. We discuss new opportunities inspired by this analogy to enhance predictive capabilities and theoretical developments in both research fields, especially using white‐box modeling for expanding predictive theory in evolutionary biology.

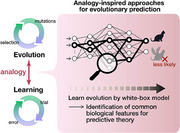

## 1 Introduction

How does the process of organismal evolution resemble “learning” in a broad sense? Although not driven by intention, organisms are engaged in a form of trial and error to find adaptive phenotypes through evolution. By creating phenotypic variations (trials) through mutations and/or phenotypic plasticity, organisms not only create abnormal and lethal phenotypes (errors) but also succeed in finding more adaptive phenotypes. Some of these variants may achieve an enhanced opportunity to survive and reproduce successfully, while others do not fare equally well [1]. Consequently, as such a process repeats across generations, this iterative cycle leads to the evolution of organisms with adaptive or fitted features or traits. This iterative nature of adaptive evolution has been noted to resemble learning processes, which similarly optimize through trial and error to find better solutions [2]. Although this conceptual concurrency has intrigued scientists ever since and has been discussed repetitively for several decades [3, 4, 5, 6, 7, 8, 9, 10, 11] (including a most recent intensive discussion on “How can evolution learn?” by Watson and Szathmáry [9]), its impact on each other's field has not reached its full potential. This is mainly due to the challenges and difficulties in formulating learning processes, leaving the analogy with unclear correspondences between the two. However, this situation is rapidly changing with the astonishing advancements in machine learning technologies, as these have started to allow scientists to replace abstract concepts of learning with more tangible and formalizable implementations [8, 12, 13].

In this review, we will seek for and highlight possible correspondences between machine learning and evolution by referring to a variety of phenomena to find hints to foster new approaches in both fields. Examples include, to name a few, the similarities between overfitting in machine learning and evolutionary trade‐offs, the parallels between the dynamics of Generative Adversarial Networks (GANs) and competition between predator and prey, historicity by training dataset and phylogenetic inertia or evolutionary bias, and so on. Such analogous relationships not only reinforce that machine learning and evolution may operate under similar principles but these could also be leveraged to develop new approaches and understandings in evolutionary studies and new implications for the design of machine learning algorithms.

Recently, an accumulating number of studies have started to utilize machine learning to identify hidden patterns and rules within data, and even to predict evolutionary outcomes [14, 15]; however, the goal of this review is not simply to reaffirm the benefits of applying machine learning in evolutionary studies. These applications, for instance, were often done by machine learning models that lack interpretability (often referred to as “black‐box” models). This lack of interpretability hinders scientists from fully understanding the underlying mechanisms and elucidating the common algorithms that drive these predictions. Consequently, each study may tend to develop an independent model, making it difficult to extract common principles that can be integrated into the extended modern synthesis. On the machine learning side, just like how organismal evolution inspired the development of the genetic algorithm (GA) [16], the potential impact of the analogy would be to bring about new algorithms for machine learning. For example, unlike machine learning, which is based on human concepts, the evolutionary process is a self‐organizing phenomenon driven by enormous numbers of living organisms that have existed in the past and present. This could lead to novel inspirations for developing new algorithms, as well as understanding how and why some algorithms show a high performance but lack an understanding of why they work that way (e.g., stochastic gradient descent [SGD]). Additionally, evolution has the potential to provide insights for algorithmic design that enable machine learning parameters to escape from a locally optimal state and transition to other states for further learning, since organisms diversified by successively shifting from one ecological niche to another.

## 2 Examples of Analogies Between Machine Learning and Evolution

Here, we will present several specific examples of analogies, ranging from classic to cutting‐edge ones, to help concretely visualize the analogous relationship between machine learning and evolution.

### 2.1 Genetic Algorithms and Darwinian Evolution

**FIGURE 1:**
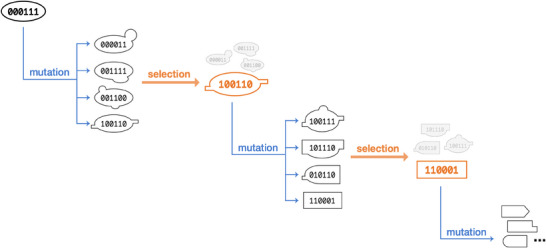
Genetic algorithm. Genetic algorithm was inspired by the idea of Darwinian selection, which solves a problem by iteratively introducing mutations and selecting the better solutions with higher fitness.

Perhaps the most well‐known example inspired by the analogy is the development of GAs and other evolutionary algorithms (EAs) [17]. GAs learn to find an optimal solution to a problem by iteratively introducing mutations into the set of possible solutions, or population, and selecting which solutions perform better [18]. These algorithms borrow the concept of fitness from evolutionary biology as an objective function to guide the optimization procedure (Figure 1). They mimic how natural selection works in principle during the learning process. In practice, these algorithms are effective especially in problems where the search space is too large for exhaustive searches, such as in exploring the parameters for merging different large language models into a new model with diverse capabilities [19]. Just as natural selection selects for better‐fitted individuals, GAs evaluate and seek for the best or quasi‐best performing solutions by introducing mutations to explore and exploit the solution space, evolving increasingly refined solutions over generations. Various ideas from evolution, such as crossover (partial mixing of solutions), were later incorporated into refined methods in GAs to improve the optimization process [17]. GAs and EAs in turn allowed evolutionary biologists to study the evolutionary origin of complex features [20], modularity [21], and even the development of cancerous tumors in the body through modeling approaches [22].

### 2.2 Overfitting and Evolutionary Trade‐Offs

**FIGURE 2:**
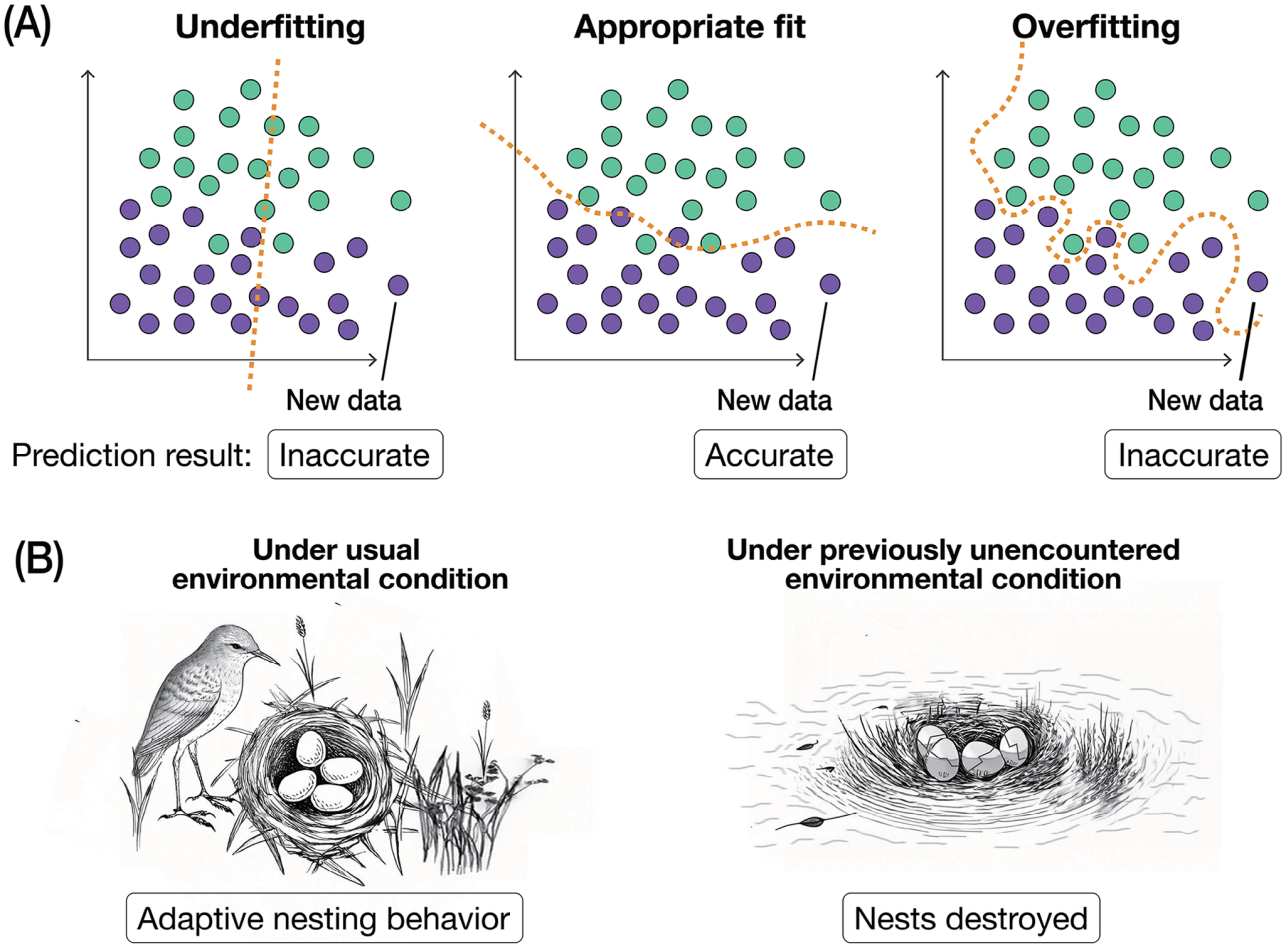
Analogy between overfitting and evolutionary trade‐offs. (A) Machine learning models are designed to find generalizable solutions when learning from the training data. In this schematic diagram, the model learns to separate two groups of data (green versus purple dots) from each other by the yellow dash line. The yellow dash line separates the two groups fairly well when the model finds a good fit to the data (middle). However, the risk of overfitting increases when the input data is insufficient, biased, or flawed. An overfit model (right) loses the ability to generalize; for example, the model may not be able to properly categorize new data properly (i.e., purple new data being misclassified as green). (B) Organisms that become too adapted to specific habitats may struggle to cope with previously unencountered environmental changes. Ground‐nesting birds, for example, are vulnerable to flooding (unencountered environmental conditions) despite the advantages of nesting at ground level.

Learning is not merely a process of memorization; it is about acquiring the ability to generalize over similar cases and make better estimations based on that. Likewise, machine learning models aim to generalize from the training data by estimating hidden patterns, enabling them to make predictions about new, unseen inputs. However, when a model becomes overly specialized to the training data, a situation called “overfitting” occurs, where the model performs extremely well only on the training data but fails to make accurate predictions when encountering new inputs [23]. In overfitting, instead of learning the general patterns, it starts to pick up specific details like noise and exceptions unique to the inputs (Figure 2A). As a result, when a model begins to overfit, it gradually loses the ability to accurately predict outcomes on new data because those specific details and noises do not apply to the unseen data. An extreme case of overfitting can occur when a model merely memorizes each input–output pair in the training data. For example, an overfit model might correctly translate every sentence from its training set into a foreign language but struggle with translating new phrases it has not encountered before. This arises because the overfit model has failed to learn the fundamental and generalizable rules of the language, such as grammar and syntax, from the training data. In more modern machine learning, especially deep learning, this understanding of overlearning is further developed [24, 25].

In the biological context, similar phenomena can be observed in organismal evolution, where an organism develops specialized traits that help it survive in a specific environment (or niche) but its ability to thrive in a broader range of environments becomes substantially lower [26, 27, 28]. One example is the vulnerability of some organisms to rare selective pressures or rare conditions in their environments that they are not adapted to handle. For instance, some ground‐nesting birds, such as certain species of sparrows and shorebirds, construct their nests close to the ground. This behavior provides multiple advantages, including camouflage from predators [29]. However, occasional flooding would destroy their nests and pose a serious threat to their reproductive success. Despite the obvious risk, many of these birds continue to nest on the ground, as their nesting behavior has been fit to ground environments, with limited plasticity to adjust to rare but catastrophic events like flooding [30, 31]. Even though nesting at higher elevations or in trees could prevent such losses, their ground‐nesting behaviors show that such birds appear to be “overfit” to their ground habitats, which makes them less capable of adapting to rarer conditions in the environments. Consequently, their reproductive success is significantly reduced when confronted with unexpected environmental changes due to such trade‐offs.

Another example analogous to overfitting would be asexual reproduction. Classic theories predicted that asexual organisms (without recombination) are quick in reaching the adaptive state in a multipeaked fitness landscape; however, they often become trapped in suboptimal adaptive peaks [32, 33]. Although asexual reproduction allows organisms to expand rapidly, they often become trapped in suboptimal adaptive conditions, making it difficult to reach the global optimal peak of the landscape [33, 34, 35]. These examples of evolutionary trade‐offs leave organisms vulnerable to new or rare environmental conditions because they become “overfitted” to the conditions they are adapted to, which resembles how an overfitted machine learning model performs well on training data but fails to generalize. An inspiration from this analogy is that understanding of how organisms overcome sub‐optimal conditions may enlighten approaches to avoid overfitting in machine learning.

### 2.3 GANs and Competition

**FIGURE 3:**
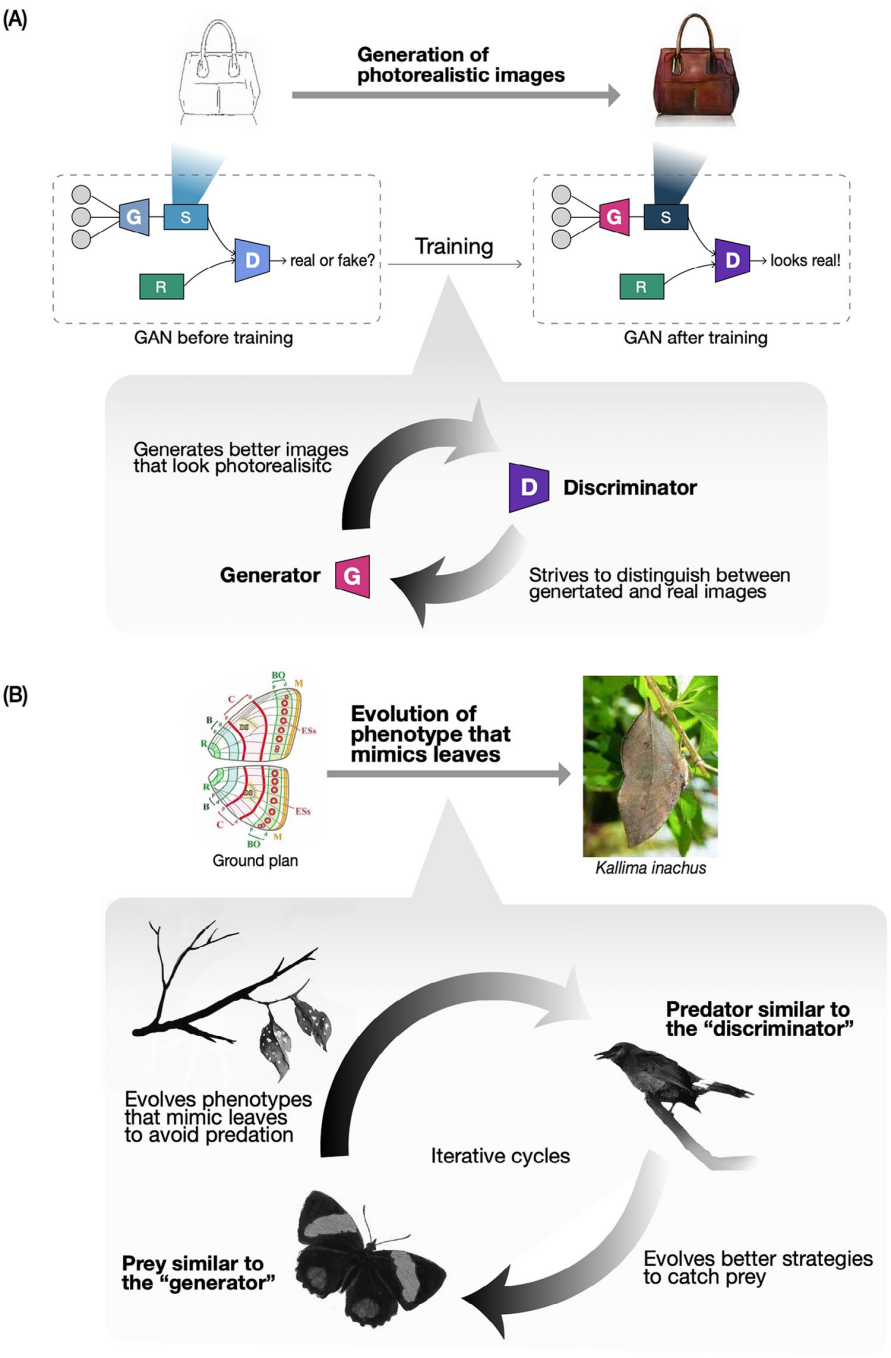
Analogy between Generative Adversarial Networks (GANs) and competition in evolution. The competitive cyclic design in GANs resembles the competitive nature between predators and prey in evolution. (A) GANs are composed of a generator [G] that creates new images and a discriminator [D] that evaluates whether the newly generated sample [S] is different from the real data [R] or not. During the training process, the generator and the discriminator strive to outcompete each other, so that the generator creates new samples that look sufficiently similar to the real data and the discriminator distinguishes the samples from the real data as much as possible. This iterative process results in models capable of creating photorealistic images, such as the handbag image (right). Illustrations of handbags are adapted from [39]. (B) In nature, predators strive to develop strategies that assist them in catching their prey whereas prey strive to avoid predation by the enemies. As a result, some species evolve morphologies that look sufficiently similar to the environment so that the predators cannot distinguish them from the surroundings. For example, the butterfly evolved a wing pattern that resembles leaves to evade from predation. Illustration of the ground plan of *Nymphalid* wing patterns and the photo of *Kallima inachus* are from [44].

Another good example of the analogy is seen in a class of machine learning models known as GANs. GANs are a kind of unsupervised learning process consisting of two major components: a “generator” that creates data, and a “discriminator” that evaluates it [36, 37, 38]. During the learning process, the generator strives to produce data that closely mimics the original training data, introducing variations that challenge the discriminator's ability to distinguish between authentic and generated data. This competitive cyclic design allows GANs to generate highly sophisticated output from the original input, such as a photorealistic image from a rough line drawing (Figure 3A) [39]. The adversarial form of the two models has been mathematically generalized, and many variants of GANs have been developed [40, 41, 42].

This “competition” between the generator and discriminator mirrors evolutionary dynamics between antagonistically interacting species, such as predators and prey, where each species evolves new adaptations across generations to outcompete the other. Often, both predators and prey coevolve rapidly in response to each other's new strategies, such as by learning to outcompete each other's movement or foraging strategies [43]. Similarly, how butterflies have evolved mimetic wing patterns may exemplify this conceptual parallel. Butterflies, as prey, strive to avoid predation from their enemies. Although ancestral *Nymphalid* butterfly wings might have a simpler ground plan, leaf butterflies (*Kallima inachus*) evolved wing patterns that closely resemble leaves [44]. To avoid being caught, the evolutionary pressure faced by these butterflies to look as similar to the surroundings as possible mirrors how GANs gradually learn to create photorealistic images from sketches (Figure 3B). In turn, predators such as birds may gradually learn and evolve better ability to distinguish mimetic butterflies, although we note the exact mechanism for this is poorly understood with a relatively evolutionarily conservative visual system in the birds [45, 46, 47, 48]. Similarly, the evolutionary arms race between a parasite or pathogen and its host species also involves such competitive or antagonistic coevolutionary dynamics. Hosts may develop rapid adaptations in their immune system to enhance pathogen detection and responses to parasitic infections. Conversely, parasites might evolve sophisticated immune evasion strategies, such as using surface and secretory proteins that mimic host molecules to avoid detection [49, 50]. Often, genes involved in predator‐prey competition become some of the fastest‐evolving genes in the genome, suggesting the effectiveness of this process during evolution [51, 52].

Such parallel dynamics highlight how the generator and discriminator in GANs and antagonistic species involve a continuous struggle to outcompete each other, where the competition drives the evolution of complex strategies and behaviors, and enhances GANs’ ability to generate convincing data. Simultaneously, this showcases the potential for new approaches being inspired by the analogy between learning and evolutionary processes to be developed, such as simulating possible evolutionary pathways of the leafy pattern from the *Nymphalid* ground plan. However, it has to be noted that mimesis evolution depends on a complex interaction between the environment and predator cognition, and it can lead to either increased or reduced polymorphism [53, 54, 55, 56, 57, 58]. This indicates that applying the analogy has to be cautious in implementing the machine learning models based on the phenomenon being studied.

### 2.4 Historicity in Machine Learning and Evolution

**FIGURE 4:**
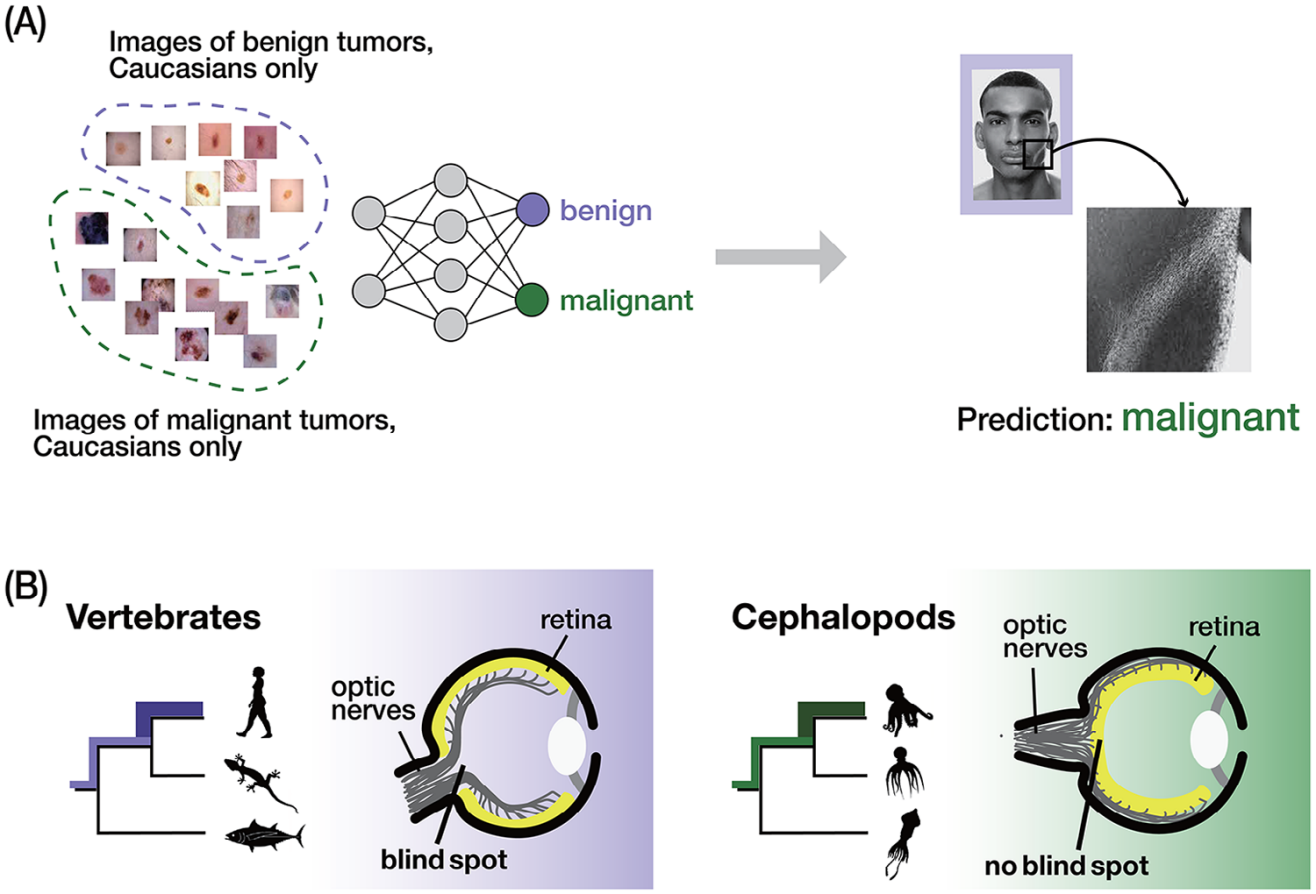
Both learning and evolution show historicity. (A) Machine learning models can be biased by training data. For example, models trained on datasets of tumor images from certain demographics may have low accuracy in predicting tumor malignancy of people from underrepresented demographics. That is, in these early models, people with dark skin were found to have higher probability to be diagnosed with malignant tumors because the training data may have been biased toward other demographics. Images are used to visualize the concept only: Tumor images are from the “Melanoma Skin Cancer Dataset” on Kaggle; the image of human face is from the “Human Faces” dataset on Kaggle as an example of a demographic underrepresented in the training dataset. (B) Vertebrate eye structure as an example of bias from evolutionary history, or historicity. Although the camera‐type eyes of both vertebrates and cephalopods are evolutionarily elaborate structures, a blind spot remained through evolution on the back side of the eyes in vertebrates which is absent in the eyes in cephalopods. This difference would be due to the different evolutionary history experienced in the cephalopod and vertebrate lineages. Animal silhouettes are from PhyloPic.

Historicity is another phenomenon that can be observed for both learning and evolution. Just as we humans can be biased by what we have learned in the past, machine learning models can be heavily influenced by their training processes and datasets, leading to biases in many different ways. An example is that if a machine learning model is only trained to recognize tumor images of certain demographics, it may perform poorly when presented with images from demographics that are underrepresented in the training data (Figure 4A) [59]. Similarly, if a model is only trained on the faces of cats, it will be biased toward cats, and that it cannot recognize dogs is not surprising at all. It is also clear that such data‐derived historicity sometimes affects the machine learning model as a bias and has a significant impact on its predictions [60].

The phenomenon of historicity can also be observed in organismal evolution. For example, some traits remain despite the fact that there could be an alternative or possibly more adaptive trait, at least hypothetically. A well‐known example is the presence of a blind spot in the vertebrate eye. Although both vertebrates and cephalopods evolved complex, camera‐type eyes independently, the different evolutionary paths of each group have led to vertebrates having a blind spot, which is absent in cephalopod eyes (Figure 4B) [61]. In vertebrates, the axons of photoreceptor cells in the retina protrude away from the incoming light, resulting in the formation of a blind spot where the optic nerve exits the eye, as no photoreceptor cells are present in that region. By contrast, cephalopods develop their eyes without a blind spot with the axons of their photoreceptor cells protruding into the direction to the brain [61, 62, 63, 64, 65]. The persistence of the blind spot in vertebrates would reflect the influence of historicity in evolution, where the evolutionary paths in the common ancestors shaped traits in the descendants, despite the fact that there could be a better solution. This is obviously similar to how learning can be biased from historical influences. Given the analogy between evolution and learning, it is inevitable that organisms show historicity to some extent, biasing what traits organisms retain and what traits organisms can evolve. Furthermore, it is anticipated how the analogy between learning and evolution can inspire insights into how biases by historicity occur.

### 2.5 Continual Learning and Exaptation

**FIGURE 5:**
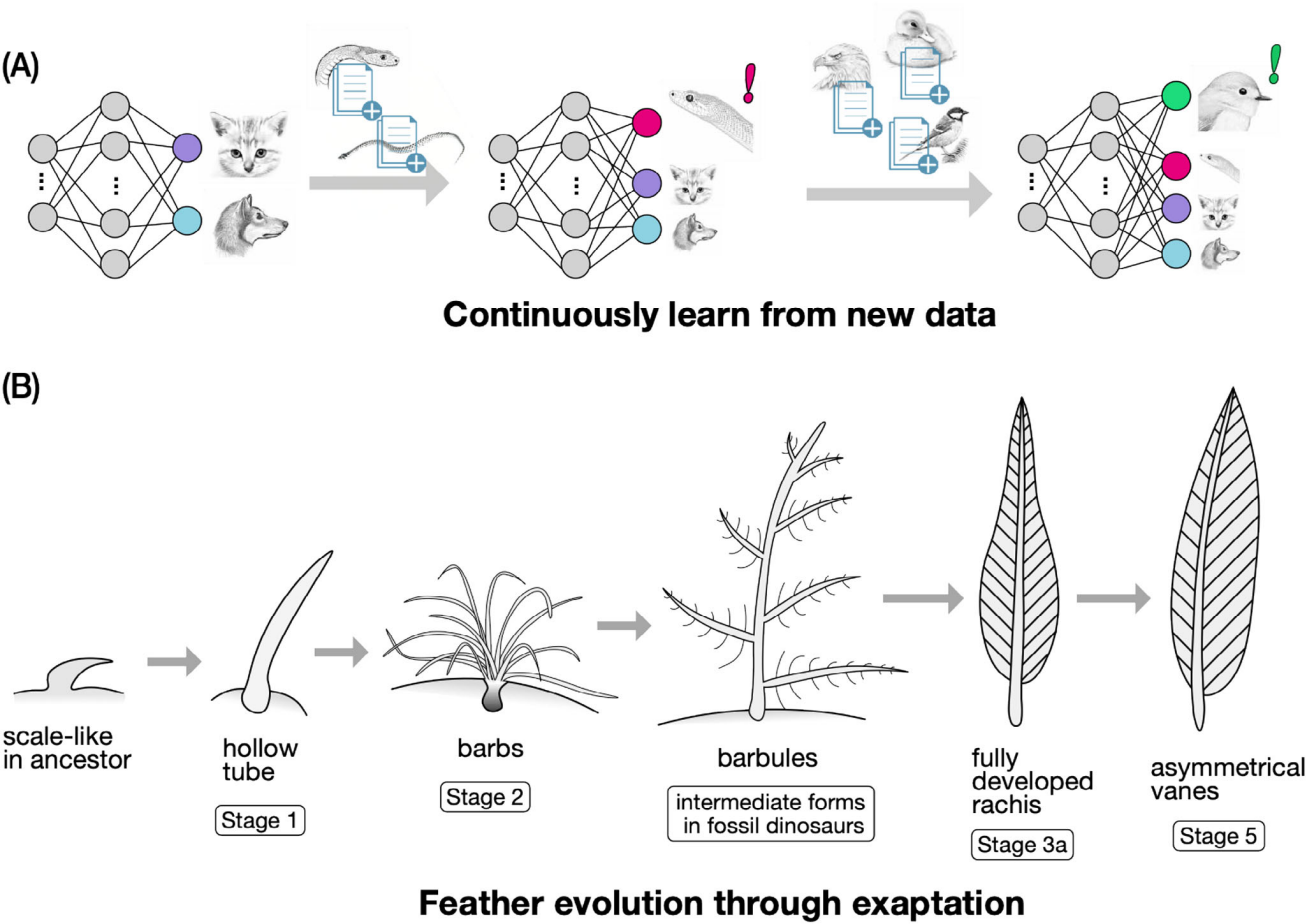
Analogy between continual learning and exaptation. Continual learning and exaptation in evolution are analogous in that both learn new things upon what they have learned previously. (A) Continual learning without forgetting previously learned knowledge is a general feature of learning by humans. Recently, it has been demonstrated that machine learning models are also able to achieve this by newly devised approaches. In continual learning, machine learning models gain the ability to predict outcomes in new tasks (highlighted by the ! marks) without losing the predictive ability in previously learned tasks. (B) Evolution achieves continuous acquisition of new adaptations. In some cases, functions of previously evolved traits may be retained. For example, it is now generally considered that feather was first evolved for thermal regulation. Although subsequent changes in the Shh‐BMP2 signaling allowed for the development and emergence of diverse feather types including those that enable flying abilities, feathers for thermal regulation (i.e., down feather) can still be seen in extant birds. Illustrations are modified based on [70, 71, 73].

Another fundamental characteristic of learning is its ability to continuously learn and adapt to new information without forgetting previously acquired knowledge (Figure 5A). Although it was once thought of as a difficult task in machine learning because older models often forget previously learned tasks rapidly when being trained to learn new tasks [66], emerging strategies have started to provide promising solutions to overcome this difficulty [67, 68]. These new approaches have started to better align machine learning models to real‐world learning behaviors and may provide insights into approaches that facilitate the retention of old skills alongside learning to perform new tasks.

Such kind of continual learning is obviously a characteristic of biological evolution, where organisms continuously evolve new adaptations often based on modifications on their existing traits. This process, known as exaptation in evolution, reutilizes old traits that were fitted to a different environment; however, the emerging new traits often show additional or different functions from the original structures. For instance, feathers of birds are thought to have originated from epidermal structures unrelated to flight in their reptilian ancestors [69, 70]. Their common ancestor was likely to have keratin‐based scales covering their skin for protection like an armor. But through evolution toward the dinosaur and avian lineage, scales gave rise to structures that initially enhanced thermal regulation, eventually evolving into the down feathers seen in extant birds. Over time, additional modifications led to the diversification of feathers, including asymmetrical flight feathers that enabled powered flight. Importantly, while feathers adapted for flight lost their original thermoregulatory function, many birds still retain down feathers for insulation alongside flight feathers. This example of birds evolving a variety of specialized feather types while preserving more ancient structures [70, 71, 72, 73, 74, 75] parallels the underlying characteristic of learning processes. Just as learning systems acquire new functions without necessarily erasing previous knowledge, avian evolution involved the addition of new feather types (such as flight feathers) while still maintaining thermoregulatory feathers. This process exemplifies how evolutionary innovations can lead to the coexistence of both novel and ancestral traits over time, which is similar to the underlying characteristic of continual learning processes.

Interestingly, as biological exaptations are considered to build new structures based on existing traits, recent studies also found molecular evidence supporting this idea. For example, it was found that the evolution of feathers likely involved the re‐purposed use of existing genes, or gene co‐option, such as Shh‐Bmp2 signaling pathways which enabled scales to evolve into highly branched scales, or feathers [71, 72]. Similarly, in the evolution in beetles, while the hindwings have been maintained for their flying ability, beetles also evolved external elytra, which are hardened wing covers that protect their bodies and aid in thermal regulation. Similar to the case of feather development and evolution, the acquisition of elytra was found to involve the co‐option of genes originally involved in exoskeleton formation [76, 77]. In both cases, these adaptations demonstrate how organisms can evolve novel traits from existing traits, sometimes by utilizing existing modules. Collectively, these processes enable organisms to adapt and evolve new functions by leveraging preexisting genetic and phenotypic structures.

### 2.6 Reinforcement Learning and Evolution to Maximize Fitness

Learning can also proceed in a way to maximize its reward for certain tasks. For example, when learning to play a video game, a person strives to achieve as high scores as possible by trying different strategies through trial and error. Having a goal to maximize reward has been found practical in recent machine learning approaches known as reinforcement learning [23, 78], and these studies have led to the powerful AlphaGo [79] that beat the top human players of Go chess in the world. Specifically, in reinforcement learning, an agent is trained to make better decisions by maximizing rewards through trial‐and‐error interactions with their environment [78]. For example, to train an agent to play a game like Mario World, at the beginning of the learning process, the agent usually loses the game very quickly because it does not know how to avoid traps and enemies. However, after repeated rounds of trials and errors, the agent will start to know how to walk, run, and “realize” that jumping to hit the bricks will get the coins and get higher scores. The agent will also gradually know where to start jumping in order to kick off the turtle enemies, avoid traps, and finally win the game. This ability is achieved by learning which decision (walk, jump, or stay still, etc.) at each time point of the game would maximize the chance to win the game and have the final reward.

The analogy in biological evolution is evident as it not only mimics the behavior of organisms but is also driven by maximizing the reward, which is similar to evolutionary processes where organismal populations tend to end up evolving or fitting into better‐fitted niches, where higher fitness is often attained. For example, the complex and beautiful patterns of train feathers in peacocks evolved because the patterns were associated with higher fitness, and the selection from females worked as feedback to evolve more beautiful patterns.

Interestingly, the aimlessness of noise added in machine learning may bear resemblance to mutations in biological evolution. During learning guided by SGD (a widely used method for optimizing loss functions), random noise is introduced into the process. Despite their apparent aimlessness, this noise often improves learning by helping models escape local minima or avoid overfitting to uninformative features [80, 81]. This is conceptually similar to mutations in evolution, which occur without specific direction but introduce variations that allow populations to explore new adaptive peaks. Despite their randomness, both noise and mutations help to navigate complex search spaces in parameter optimization or evolutionary fitness landscapes.

In addition, in reinforcement learning, when deciding the next actions to achieve higher reward, the learning agents often face the conflicting problem of whether to explore new strategies more, or explore less but instead exploit current knowledge to achieve the highest possible reward based on what it has already learned. The benefit of explorations in learning processes is similar to how increased mutations may lead to the generation of more variable phenotypes for selection, whereas mutations may also cause harmful or lethal traits to the organisms. These similarities suggest that learning and biological evolution in general may share a lot of underlying principles, and in addition, insights from each side may also inspire the other field to achieve a better understanding of how the learning process or evolutionary process actually works.

### 2.7 Differences Between Learning and Evolutionary Processes

We have explored six concrete examples to illustrate the analogous relationship between (machine) learning and evolution above. Meanwhile, it is noteworthy that differences do exist between the two, especially because a wide variety of new techniques and artificial optimizations to improve algorithmic efficiencies have been introduced [82, 83]. Essentially these do not have direct counterparts in nature. For instance, in modern GAs, mutations and recombinations are designed to efficiently search the solution space and optimize performance as rapidly as possible. These artificially engineered mechanisms enhance computational efficiency but do not necessarily reflect the stochastic and potentially constrained nature of evolutionary processes in biological systems. Thus, caution is required when drawing corresponding counterparts between GAs and biological evolution. Similarly, in GANs, although the competitive dynamic between the generator and discriminator is conceptually similar to coevolutionary interactions between predators and prey, GANs differ from organismal relationship since developments of techniques to enable accurate predictions and efficient generation of photorealistic images with high speed have been introduced [84, 85]. Another point that is also noteworthy is that while machine learning aims to actively seek for ways to lower the loss function or to achieve higher rewards (such as in reinforcement learning), biological organisms do not have a specific aim in evolution. These suggest that while machine learning and biological evolution share many conceptual similarities, caution is needed when conducting studies based on the analogy.

## 3 White‐Box and Explainable Machine Learning as Potential Tools to Expand Modern Evolutionary Theories

### 3.1 Applying the Analogy to Predict Evolution

Given the analogous relationship between learning and evolutionary processes discussed in section 2, one exciting implication is that models trained to learn the evolution of biological organisms or their phenotypic evolution may be able to predict their evolutionary outcomes. Although several pioneering studies have attempted to devise new approaches inspired by the analogy to model evolution (such as to utilize formulations of machine learning process to model biological evolution in Vanchurin et al. [12], and to model cancer evolution in Lahoz‐Beltra et al. [22]), how the analogy can be operationalized to predict various evolutionary phenomena remains largely unexplored. This idea echoes a long‐standing challenge in evolutionary biology, as evolution has often been viewed as a study of biological histories whereas predictive theories of contemporary and future evolution, especially those predicting how phenotypes evolve, remain scarce. Further research is awaited to investigate which evolutionary phenomena are more effectively analyzed using such analogy‐based approaches.

In particular, it remains largely unknown and underexplored what kind of common mechanisms can be found for predicting evolutionary outcomes. In this regard, even though discussions about the predictability of evolutionary outcomes have surfaced sporadically [86, 87, 88], only recently have studies begun to propose and support potential mechanisms that govern phenotypic evolution. For example, phenotypes expressing many pleiotropic genes (those used in multiple developmental processes) [89] and exhibiting high developmental stability (embryos with fewer fluctuations in gene expression level) [90, 91] correlated with its evolutionary conservation. Although we anticipate that further utilizing these clues may help the development of predictive theories of evolution, it is possible that the analogy can be utilized to uncover biological features that are useful in understanding the common mechanisms in predicting phenotypic evolution. In other words, given the analogy between learning and evolution, understanding how machine learning models predict phenotypic evolution may reveal the common mechanisms to predict biological evolution.

### 3.2 Limitations of Current “Black‐Box” Machine Learning Approaches

**FIGURE 6:**
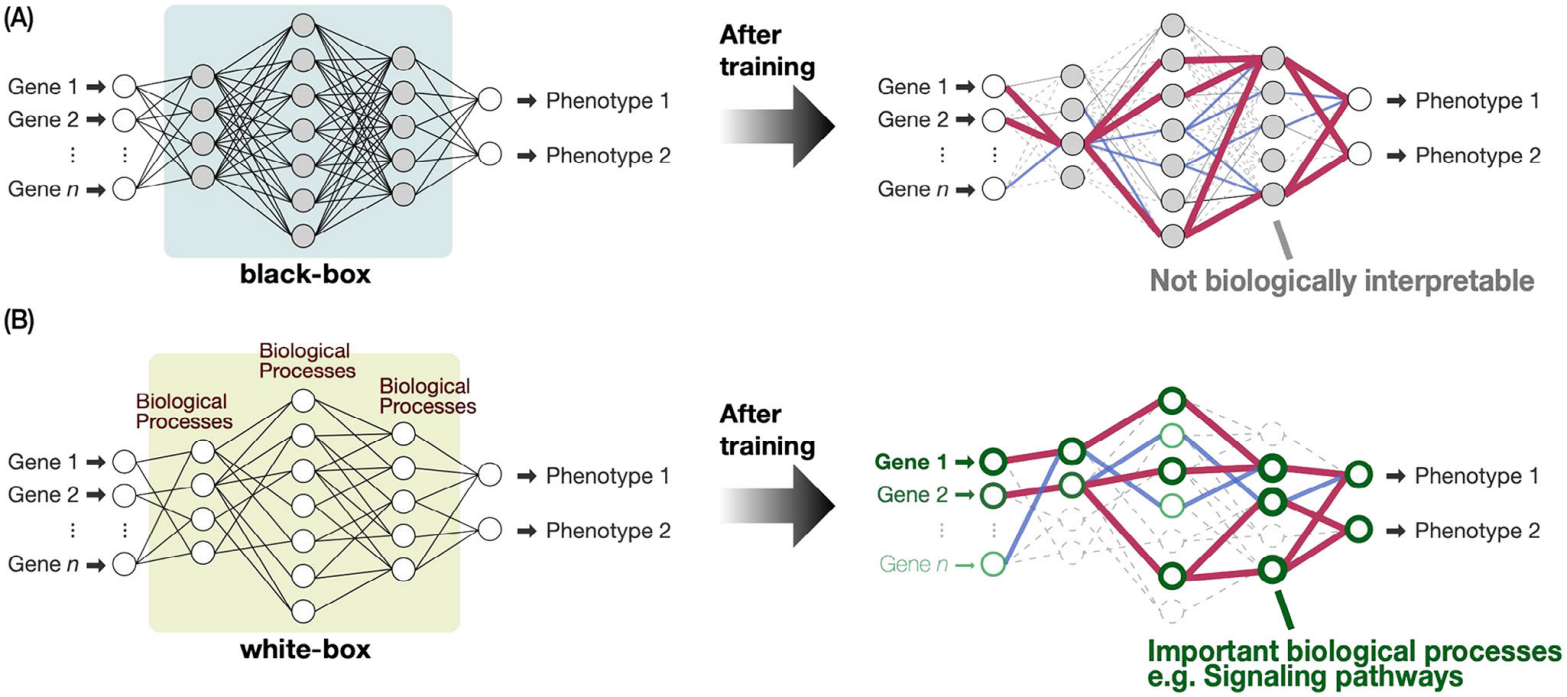
White‐box modeling enables the interpretability of predictions. (A) Schematic illustrations of black‐box machine learning models. Even though high accuracy of predictions can often be achieved, it remains difficult to understand why the model makes certain predictions. Nodes that are not directly interpretable in the intermediate layers are colored in gray. (B) By contrast, white‐box modeling allows for higher interpretability of predictions because the architecture of a white‐box model is formed by nodes and edges that are biologically interpretable (e.g., representing known biological processes). By utilizing explainable machine learning techniques, it becomes possible to trace which nodes and edges (and therefore, which biological processes) are important for making predictions. As an example, important nodes are highlighted in darker green colors. Edges (relationships between biological processes) colored in magenta are more important for making predictions than the ones in blue. Nodes and edges marked by grayish dashes contribute less to the prediction output.

Importantly, although pioneering attempts in predicting phenotypic changes have started to emerge in recent years, the progress is still largely confined to specific cases, such as artificial selection in agricultural crops [92, 93] and the evolution of antibiotic resistance in bacteria [94, 95]. A major limitation is that such models may only be applicable to specific cases, making it difficult to derive general laws or biological principles that could contribute to a broader predictive framework in evolutionary biology (e.g., to expand the extended modern synthesis). This is due to the difficulty in understanding how black‐box machine learning approaches predict. For instance, machine learning was used to predict which variants of human influenza viruses are more likely to persist and dominate in the near future [14]; however, even though the prediction did not involve a highly complex model, elucidation of the reason why the predictions were accurate or trustworthy remained difficult (Figure 6A). Although achieving high accuracy of prediction has excited many scientific disciplines, explainability of machine learning models in biological terms remains a major barrier for scientists to further expand the findings into a common mechanism behind these, or to formulate a general theory of evolutionary prediction. Therefore, in leveraging the analogy between learning and evolution to predict biological evolution, an important consideration is not only whether the model can predict evolution but also we can understand the algorithmic logic and extract features behind how the evolutionary predictions are made in a biologically interpretable way.

### 3.3 White‐Box Models Have the Potential to Extract Common Biological Features Driving Predictions

To overcome the limitations of black‐box models, recent studies have set out to look for approaches that support interpretability in biological terms while simultaneously being able to achieve high accuracy in prediction. This has brought a new, contrasting approach called “white‐box” modeling to growing attention [96]. Unlike black‐box models, where their internal decision‐making processes are often obscured (Figure 6A), white‐box models are transparent and interpretable. White‐box models are often made up of nodes that are interpretable, and the connections between the nodes could be imposed by explicit rules (Figure 6B) (such as models imposed by physics rules in physics‐informed machine learning models [97]). In other words, such design in the machine learning model architecture allows their inner workings to be understood. Given the analogy between learning and evolution, our speculation is that such a biologically interpretable machine learning approach may not only allow for predictions of evolutionary outcomes but can also enable the identification of key biological features that drive those predictions.

In practice, while still being an emerging approach, such biologically interpretable or biologically informed neural networks have started to bring about new discoveries in other fields of biological sciences. These pioneering studies utilized biologically informed models composed of nodes representing known biological pathways or processes, such as those representing gene functions, Gene Ontology, or KEGG pathways. After training the model, nodes and connections in the neural network (which correspond to specific biological functions) that are important for making the prediction can be identified by explainable machine learning techniques (Figure 6B). To note, in biomedical sciences, such transparency in machine learning models is particularly high in clinical applications because doctors are often unable to tell why certain predictions can be made by machine learning software, making it impossible to evaluate whether the prediction is trustworthy or not (such as in judging whether a radiological image shows the signs of cancer) [98, 99]. These urgent needs have called for the rapid development of white‐box and explainable machine learning approaches, and some pioneering studies have shown that such approaches could identify previously unknown but biological factors (such as genes) important for making biomedical predictions [100, 101, 102, 103, 104, 105].

For instance, the study by Elmarakeby et al. developed a white‐box model for predicting prostate cancer from the patients’ omics profiles and they successfully identified new predictor genes and biological pathways for the disease [101]. In this study, a neural network comprised of known biological pathways was trained to learn the characteristics of omics data from healthy and cancer patients. By analyzing which components in the network drive the predictions, this led to the discovery of a previously unknown marker MDM4 and its related biological pathway as novel clinical predictors of prostate cancer [101]. Specifically, the biological network information in the Reactome database was first transformed into a neural network architecture. Next, during the training phase, only the edges (connections in the neural network) representing known pathway relationships could be trained to reveal which nodes are important in making the prediction [101]. Although this seems to limit which network components can be trained, interestingly in this study, the model achieved higher prediction accuracy compared to other dense and black‐box models, although whether high prediction accuracy is a general trend requires further investigation. Likewise, this strategy has been deployed in proteomics and epigenetics to identify key proteins associated with disease states [103] and candidate pathways that explain the epigenetic clocks [105]. Moreover, to account for incomplete biological knowledge in the databases and network structures, an improved strategy was devised in Lotfollahi et al. to address this limitation, using a flexible encoder‐decoder architecture that allows for trimming and modification of pathways when the machine learning model learns which biological pathways may underlie the identity of specific cell types based on their single‐cell expression profiles [102].

Therefore, it becomes increasingly tempting to utilize these explainable white‐box modeling and devise innovative approaches inspired by the machine learning–evolution analogy to expand the current approaches on evolutionary prediction. Importantly, this goes beyond mere prediction as the inner workings of the prediction model may help us understand the underlying mechanisms for major evolutionary trends. Without interpretability in the prediction models, even if we train models using sufficient amounts and highly diversified data from millions of species, black‐box models still do not allow us to expand current evolutionary theories because the common tendencies or rules underlying evolution cannot be extracted from such models for specific cases. That is, each black‐box model may perform very well in predicting the evolutionary outcomes, but it may only be applied in the specific cases because no rules commonly underlying different cases can be extracted. By contrast, the white‐box architecture may allow us to extract the common algorithmic logic driving the predictions from different models. Such common features or algorithmic logic may provide new hypotheses that are testable and may be used to extend evolutionary theories. Considering this as an emerging approach, it is important for future studies to start accumulating evidence whether predictions inspired by the analogy can extract common biological features that correlate with or potentially contribute to predicting evolutionary outcomes. Although it is impossible to directly test predictions that happen millions or billions of years from now on, cases of more rapid evolution or those of shorter timescales could be the starting points of such studies. By repetitively trying out such predictions, biologists would be able to start to identify which common biological features can make reliable predictions.

### 3.4 Limitations of the Analogy for Predicting Evolution

Although the analogy between learning and evolution offers a promising framework for introducing predictive theories in evolutionary biology, it is important to note that additional efforts are required to effectively apply this analogy to evolutionary studies.

A primary challenge lies in operationalizing this analogy to extract common biological mechanisms for predicting evolutionary outcomes. Although the conceptual analogy has been extensively discussed for decades, and we have explored additional concrete examples comparing with machine learning, how to practically implement white‐box approaches that can learn evolution from empirical data needs further studies. In particular, technical challenges are often confronted when bridging the gap between machine learning's engineering demands and biological data. These include, for example, the limited quantity of biological experimental data for training, the scarcity of appropriate datasets for validating machine learning‐derived long‐term evolutionary predictions, and the difficulty in constructing hierarchical parameters with biological reliability. Pioneering studies in other biological fields that made the first attempts to devise practical white‐box machine learning approaches would provide hints for overcoming these challenges (e.g., Yang et al. *Cell* 2019 [100]; Elmarakeby et al. *Nature* 2021 [101]; Lotfollahi et al. *Nature Cell Biology* 2023 [102] that uncovered previously unknown biological networks underlying antibiotic actions, cancer, or cell types by using white‐box machine learning).

## 4 Conclusions

Although the analogous relationships between learning and evolutionary processes have been discussed over decades, we expanded these using specific examples from recent advancements in machine learning and evolutionary science. These include overfitting and evolutionary trade‐offs, similar iterative nature shared by competition in evolution and GANs, historicities in both processes, continual learning and exaptation, and higher reward in reinforcement learning and maximizing fitness in evolution. Importantly, while these specific examples are expected to inspire both fields, such as providing new research strategies, the primary importance of the analogy relationship does not lie in the mere application of machine learning. Especially in the field of evolutionary biology, which primarily focuses on past events, the utilization of this analogy is anticipated to introduce a theoretical framework for predicting evolutionary outcomes in various phenomena. This could be achieved particularly through the use of interpretable machine learning approaches, such as white‐box machine learning. This is because interpretable machine learning allows scientists to identify common factors and mechanisms behind the predictions of different evolutionary phenomena, whereas the mere application of black‐box machine learning often leads to case‐specific predictions.

## Author Contributions

Conceptualization: Jason Cheok Kuan Leong and Naoki Irie. Writing: Jason Cheok Kuan Leong and Naoki Irie wrote the initial draft with substantial input from Masaaki Imaizumi and Hideki Innan. All authors have agreed to the submitted and revised versions. Figures: Jason Cheok Kuan Leong and Naoki Irie with substantial input from Masaaki Imaizumi and Hideki Innan. Supervision: Naoki Irie. Funding acquisition: all authors.

## Notes on the Use of AI in the Manuscript

ChatGPT (GPT‐4o) was utilized to improve the phrasing and word choices of the manuscript. Perplexity Pro and SciSpace were used to collect research articles related to the topics, in addition to a more manual literature search using Google Scholar, PubMed, and Web of Science. Literature collected by the AI tools was thoroughly read and investigated by all authors, and only those that properly support the arguments were cited. Despite the involvement of various AI tools in improving the manuscript, the entire conception of the manuscript and the arguments are originally from the authors. Parts of the illustrations are from online resources including PhyloPic, Wikimedia Commons, Kaggle, as well as from published research articles based on CC BY 4.0. To illustrate the abstract concepts presented, the sketch drawings in Figures 2, 3, and 5A were created with assistance from Adobe Photoshop and Firefly (generative AI) and were guided by images of relevant species from the Internet. The images were then further modified manually by the authors to ensure biological accuracy.

## Conflicts of Interest

The authors declare no conflicts of interest.

## Data Availability

Data sharing is not applicable to this article, as no new data were created or analyzed in this study.
